# From Membrane Destabilisation to Metabolic Collapse: Bioherbicidal Effects of *Santolina chamaecyparissus* L. Essential Oil on *Bidens pilosa* L.

**DOI:** 10.3390/biology15181606

**Published:** 2026-09-11

**Authors:** Emanuela Talarico, Eleonora Greco, Francesco Guarasci, Marina Camoli, Leonardo Bruno, Fabrizio Araniti

**Affiliations:** 1 Unit of Plant Biology, Department of Biology, Ecology and Earth Sciences (DiBEST), University of Calabria, 87036 Arcavacata of Rende, Italy; emanuela.talarico@unical.it (E.T.); eleonora.greco@unical.it (E.G.); francesco.guarasci@unical.it (F.G.); marina.camoli@unical.it (M.C.); leonardo.bruno@unical.it (L.B.); 2 Department of Agricultural and Environmental Sciences—Production, Landscape, Agroenergy (Di.S.A.A.), University of Milano, 20133 Milano, Italy

**Keywords:** natural herbicide, essential oil, membrane disruption, photosystem II, oxidative stress, metabolomics

## Abstract

The increasing incidence of herbicide-resistant weeds and concerns over synthetic herbicides are driving the search for natural alternatives. This study evaluated the post-emergence bioherbicidal activity and mode of action of *Santolina chamaecyparissus* L. essential oil against *Bidens pilosa* L. seedlings. Gas chromatography–mass spectrometry revealed a chemically complex oil dominated by oxygenated monoterpenes, including cuminic alcohol, cis-carveol, cis-pinocarveol, α-phellandrene, and limonene. A preliminary dose–response assay identified an ED_50_ of 0.82% (*v*/*v*). At this concentration, visible injury reached 75.9% at 72 h, while leaf relative water content declined to 40.0%. Electrolyte leakage reached 77.6% at 24 h, accompanied by a threefold increase in malondialdehyde, indicating severe membrane damage and lipid peroxidation. Chlorophyll fluorescence imaging showed impaired photosystem II function, reduced electron transport, altered energy dissipation, and loss of photosynthetic vitality. Photosynthetic pigments declined after 24 h, with chlorophyll a reduced by approximately 32.5%. Untargeted metabolomics revealed time-dependent depletion of soluble sugars, organic acids, ascorbate, and shikimate, together with accumulation of proline, γ-aminobutyric acid, trehalose, and branched-chain amino acids. Network analysis indicated loss of metabolic coordination. Overall, under the controlled experimental conditions used here, *S. chamaecyparissus* essential oil exhibited a rapid contact-type phytotoxic action characterised by membrane disruption, oxidative damage, dehydration, photosynthetic impairment, and extensive metabolic perturbation.

## Simple Summary

Weeds that survive conventional herbicides are an increasing challenge for agriculture. This study tested whether the essential oil of *Santolina chamaecyparissus* L., a Mediterranean aromatic plant, could control *Bidens pilosa* L., a common and difficult weed. The treatment rapidly damaged the leaves, caused water loss and drying, and reduced the plant’s ability to use light for growth. It also disrupted the normal balance of sugars, organic acids and amino acids, showing that the damage extended beyond the leaf surface and affected the plant’s basic metabolism. These results indicate that Santolina essential oil could be developed as a fast-acting natural weed-control product. Further studies are needed to confirm its safety for crops and the environment and to determine whether it remains effective under field conditions.

## 1. Introduction

Weed management remains a major challenge for agricultural production. Synthetic herbicides have provided effective and economically sustainable weed control for several decades. However, the recurrent use of a restricted number of herbicidal modes of action has imposed strong selection pressure on weed populations, leading to the evolution of target-site and non-target-site resistance mechanisms and progressively reducing the effectiveness of several widely used active ingredients [1]. At the same time, concerns regarding environmental contamination, effects on non-target organisms, and the increasing regulatory costs associated with the development of new synthetic compounds have stimulated interest in alternative weed-management tools. In this context, plant-derived natural products represent a valuable source of structurally diverse phytotoxic molecules and potential lead compounds for the discovery of herbicides with novel or multiple biological targets [2,3,4].

Essential oils are complex mixtures of volatile and lipophilic secondary metabolites, principally monoterpenes, sesquiterpenes, phenylpropanoids, and their oxygenated derivatives. Many of these compounds can inhibit seed germination, seedling establishment, and vegetative growth [5,6]. These properties have stimulated interest in essential oils as potential botanical herbicides, particularly for non-selective or post-emergence applications [5,6,7]. Their biological activity may depend on interactions among multiple constituents and can be influenced by chemical composition, formulation, plant developmental stage, and environmental conditions [6,7].

The herbicidal effects of essential oils frequently develop as rapid contact injury. Their lipophilic constituents can penetrate the cuticle and partition into cellular membranes, altering membrane organisation, permeability, and ion homeostasis [8]. These primary disturbances may trigger electrolyte leakage, oxidative stress, lipid peroxidation, loss of cellular water, and tissue desiccation. Damage may subsequently spread to the photosynthetic apparatus, causing reductions in chlorophyll content, photosystem II quantum efficiency, electron transport, and the capacity for regulated thermal dissipation [9,10]. However, the effects of essential oils are not necessarily restricted to nonspecific membrane disruption. Physiological and metabolomic studies have demonstrated that they may also interfere with central carbon and nitrogen metabolism, photorespiration, amino-acid homeostasis, antioxidant metabolism, and osmotic adjustment [11,12]. Integrating membrane, photosynthetic, biochemical, and metabolomic responses is therefore essential for distinguishing early primary injury from downstream metabolic consequences and for establishing a biologically plausible sequence of events underlying phytotoxicity.

*Santolina chamaecyparissus*, commonly known as cotton lavender, is an aromatic Mediterranean subshrub belonging to the Asteraceae family. Species of the genus *Santolina* have traditionally been used for their antimicrobial, anti-inflammatory, insect-repellent, and other biological properties, which have been associated with their abundance of volatile terpenoids and other specialised metabolites [13]. The essential oil of *S. chamaecyparissus* is generally rich in monoterpenes and oxygenated monoterpenes, although pronounced qualitative and quantitative variability has been reported among accessions. Profiles dominated by artemisia ketone, camphor, 1,8-cineole, cubenol, phellandrene, or other mono- and sesquiterpenoid constituents have been described depending on geographical origin, plant organ, developmental stage, harvesting period, and extraction procedure [14,15,16,17]. Consequently, chemical characterisation must accompany each biological evaluation because the efficacy and mode of action of the oil may vary substantially among chemotypes.

Although the antimicrobial and pharmacological properties of *S. chamaecyparissus* have been extensively investigated, its herbicidal potential remains comparatively less characterised. Previous studies have shown that its essential oil can inhibit the germination or growth of several weed species and may exert different levels of selectivity depending on the target plant and applied concentration [7]. However, much of the available evidence has been obtained from germination assays, early seedling tests, or experiments focused primarily on growth inhibition. The sequence linking the initial interaction of the oil with exposed leaf tissues to membrane failure, photosynthetic dysfunction, oxidative injury, dehydration, and reorganisation of primary metabolism in established weeds has not yet been fully resolved.

*B. pilosa* is an annual broadleaf species of the Asteraceae family that has become a troublesome weed in tropical, subtropical, and warm-temperate agroecosystems. Its successful establishment is favoured by prolific seed production, efficient dispersal, broad germination requirements, and the ability to emerge under a wide range of environmental conditions. These characteristics make its management particularly difficult in conservation and reduced-tillage systems, where seeds remain concentrated near the soil surface and light can stimulate germination [18]. Control is further complicated by the occurrence of populations resistant to acetolactate synthase-inhibiting herbicides and glyphosate [19,20]. Consequently, *B. pilosa* represents an appropriate target species for evaluating new natural post-emergence herbicidal products. Its sensitivity to essential-oil-based treatments has already been demonstrated for *Carlina acaulis* L. essential oil, which induced leaf necrosis, water-status alterations, photosystem II impairment, and extensive metabolic reprogramming [12].

Against this background, the present study investigated the post-emergence bioherbicidal activity and mode of action of *S. chamaecyparissus* essential oil against *B. pilosa*. The essential oil was first characterised by gas chromatography–mass spectrometry, after which its concentration-dependent phytotoxicity was evaluated to estimate the half-maximal effective concentration. The temporal progression of injury at the selected concentration was then examined by integrating visual phytotoxicity and leaf relative water content with measurements of membrane integrity, lipid peroxidation, photosynthetic pigments, and chlorophyll a fluorescence. Untargeted GC–MS metabolomics and Debiased Sparse Partial Correlation network analysis were further employed to identify time-dependent changes in primary metabolism and metabolic coordination. We hypothesised that *S. chamaecyparissus* essential oil acts primarily as a rapid contact bioherbicide, initiating membrane destabilisation and oxidative damage that lead to water loss, photosynthetic failure, depletion of carbon and antioxidant resources, and ultimately a loss of coordinated metabolic homeostasis.

## 2. Materials and Methods

### 2.1. Plant Material, Essential Oil Extraction, and Characterization

*Santolina chamaecyparissus* L. plants were obtained from a local certified commercial nursery and subsequently cultivated under field conditions at the Experimental Farm of the Università Mediterranea di Reggio Calabria, Reggio Calabria, Italy. The plant material was therefore derived from cultivated commercial stock rather than from a spontaneous local population. Aerial parts of *S. chamaecyparissus* adult plants were collected in June 2019, during the flowering developmental stage. After collection, the aerial parts were shade-dried under room temperature conditions until essential oil extraction.

Dried aerial parts of *S. chamaecyparissus* were subjected to hydrodistillation using a large-scale laboratory Clevenger-type apparatus equipped with a 12 L distillation flask. The plant material was immersed in distilled water (1:10 *w*/*v* ratio) and distilled continuously for 4 h until no further oil recovery was observed. The obtained essential oil (EO) was separated from the aqueous phase, dried over anhydrous sodium sulfate (${Na}_{2}{SO}_{4}$), filtered, and stored in amber glass vials at 4 °C in the dark until subsequent chemical characterization and bioassays. The EO extraction yield was 0.45% (*w*/*w*, dry-weight basis), calculated as the mass of recovered essential oil relative to the dry mass of the plant material subjected to hydrodistillation.

A single EO batch, obtained from the same plant-material collection and hydrodistillation procedure, was used throughout the entire study, including the preliminary dose–response assay and all subsequent physiological, biochemical, chlorophyll fluorescence, and metabolomic experiments. This experimental design ensured that all biological responses were associated with the same chemically characterized EO batch. However, because independent EO batches were not produced and compared, batch-to-batch compositional variability was not assessed in the present study.

The quantitative and qualitative phytochemical profile of the *Santolina* EO was characterized using Gas Chromatography-Mass Spectrometry (GC-MS). One microliter of EO diluted in n-hexane (1:100 *v*/*v*) was injected in split mode (split ratio 1:50). Separation was achieved using a 5-MS low-polarity capillary column (30 m × 0.25 mm i.d. × 0.25 μm film thickness), based on a 5% phenyl–95% dimethylpolysiloxane stationary phase. Helium was employed as the carrier gas at a constant flow rate of 1.0 mL min^−1^. The oven temperature program was initiated at 60 °C for 5 min, increased at a rate of 3 °C min^−1^ up to 240 °C, and finally held for 10 min. The injector and transfer line temperatures were maintained at 250 °C and 280 °C, respectively. Mass spectra were recorded in the electron ionization (EI) mode at 70 eV, scanning a mass range of *m*/*z* 40–450. Individual volatile constituents were identified by comparing their retention indices (RI), calculated relative to a homologous series of n-alkanes (C_8_–C_30_; Sigma-Aldrich, Milano, Italy) according to Davies [21], with those reported in the literature, as well as by matching their mass fragmentation patterns with reference libraries including the NIST and Wiley databases [22]. The complete commercial alkane mixture was used as supplied, although only the homologues encompassing the retention range of the detected EO constituents were required for RI calculation. The relative chromatographic abundance of each detected constituent was calculated as the integrated peak area of each constituent divided by the total integrated peak area, and expressed as an uncorrected relative peak-area percentage. Compound-specific calibration curves or relative response factors were not applied; therefore, these values should be interpreted as relative chromatographic abundances rather than absolute or semi-quantitative concentrations.

### 2.2. Target Weed Cultivation

Seeds of *Bidens pilosa* L. were collected in Palizzi (RC), Italy. The identity of the plant from which they were collected was taxonomically confirmed, and a voucher specimen (Voucher No. 21293) was deposited at the herbarium of the University of Calabria. Seeds were subsequently sown in plastic pots (10 cm diameter) filled with a standardized commercial substrate (soil:peat:perlite, 3:1:1 *v*/*v*/*v*). Plants were grown in a controlled greenhouse environment under a 16 h light/8 h dark photoperiod, a temperature regime of 25/20 ± 2 °C (day/night), and a relative humidity of 60 ± 5%. All downstream experimental steps, including preliminary screening and specialised physiological assays, were initiated when the seedlings reached a uniform 4-true-leaf phenological stage.

### 2.3. Preliminary Dose–Response Screening and ED50 Determination

To determine the optimal phytotoxic concentration of *S. chamaecyparissus* essential oil (EO) for the subsequent biochemical and biophysical investigations, a preliminary post-emergence dose–response screening was carried out. Seedlings of *B. pilosa* were subjected to seven distinct concentrations of the EO emulsified solution: 0.0% (negative control), 0.125%, 0.25%, 0.5%, 1.0%, 2.0%, and 4.0% (*v*/*v*). To facilitate EO dispersion in the aqueous phase and improve wetting of the lipophilic leaf surfaces, all solutions were supplemented with 0.1% (*v*/*v*) Osmosis (Del Bon, Udine, Italy) commercial organic adjuvant.

Immediately before application, the EO was added to the aqueous phase containing 0.1% (*v*/*v*) Osmosis, and the mixture was vortex-mixed at maximum speed for 5 min until a macroscopically homogeneous emulsion was obtained. The treatment solutions were freshly prepared for each application and were used immediately after homogenisation to minimise phase separation. The emulsion was re-agitated immediately before transfer to the sprayer and, when necessary, during spraying. No treatment solution was stored for subsequent application.

Because the emulsions were prepared immediately before use and were not stored, their long-term physicochemical stability was not investigated. No formal measurements of droplet-size distribution or time-dependent emulsion stability were performed.

Foliar treatments were applied once with a hand-held laboratory pressure sprayer, following the previously described procedure for *B. pilosa* at the four-true-leaf stage [12]. A fixed volume of 10 mL of treatment solution was applied per pot, ensuring uniform coverage of the aerial plant surface with fine droplets immediately before runoff. The same application volume and spraying procedure were used for all EO concentrations and for the corresponding negative control. Each treatment group consisted of five independent biological replicates (n=5), with pots arranged in a completely randomised design.

Visual phytotoxicity was assessed directly on intact plants by visual inspection at 24 h post-application using a continuous percentage scale ranging from 0% (healthy plant, no visible injury) to 100% (complete leaf desiccation, extensive necrosis, or structural seedling collapse). Intermediate scores were assigned according to the overall extent and severity of visible symptoms, including chlorosis, wilting, loss of leaf turgor, tissue desiccation, and necrosis, relative to untreated control plants. The preliminary screening was designed for quantitative visual scoring, and standardized photographic documentation of each individual EO concentration was not acquired. The mathematical relationship between the progressive EO concentrations and the corresponding phenotypic injury percentage was modelled by fitting the raw experimental dataset to a non-linear four-parameter log-logistic regression curve using the following equation [23,24]:

$$Y=C+\frac{D-C}{1+exp\;exp\;\left(B\times \left(log\;log\;\left(X\right)\;-log\;log\;\left({ED}_{50}\right)\;\right)\right)\;}$$

where Y represents the percentage of visual phytotoxic injury; X is the specific essential oil concentration (*v*/*v*); C is the lower response limit (asymptote representing the negative control, fixed at 0%); D is the upper response limit (maximum achievable injury asymptote, fixed at 100%); B is the slope of the curve around the inflection point; and ${ED}_{50}$ represents the effective dose required to induce a 50% visual phytotoxic or desiccating effect on the target weed population.

The non-linear regression analysis and the computation of the exact ${ED}_{50}$ value, along with its 95% confidence intervals, were calculated using the drc statistical package according to the optimization protocols described by Ritz et al. [25]. Based on the calculated inflection point of this regression model, a single optimal concentration corresponding to the ${ED}_{50}$ value was selected and systematically applied to all subsequent experimental cohorts to standardize the investigation of downstream membrane disruption, photosynthetic degradation, and metabolomic primary pathway shifts.

### 2.4. Downstream Experimental Design and Core Treatments

Following the determination of the ${ED}_{50}$ for the EO, a second large-scale standardized experiment was established using the calculated ${ED}_{50}$ dose. Plants were arranged in a completely randomized design with five biological replicates (n=5) per group. The experimental treatments consisted of:

1. Negative Control (Ctrl): Foliar application of distilled water supplemented with 0.1% (*v*/*v*) Osmosis commercial organic adjuvant, used to establish the baseline for healthy plant physiology.
2. Essential Oil Treatment (EOT): Foliar application of the *S. chamaecyparissus* essential oil emulsifiable solution prepared at the pre-calculated ${ED}_{50}$ concentration, supplemented with 0.1% (*v*/*v*) Osmosis adjuvant.

Foliar applications were performed uniformly until complete runoff and the EO emulsion was freshly prepared and homogenised immediately before application, following the procedure described in Section 2.3. All subsequent biophysical, physiological, and biochemical parameters were characterised simultaneously shortly before destructive tissue sampling.

### 2.5. Phenotypic Injury and Relative Water Content (RWC)

Visual phytotoxicity was assessed at 2, 6, 24, 48, and 72 h post-treatment on the core experimental groups. Phytotoxic injury was scored using a standardised percentage scale ranging from 0% (no visible damage/healthy plant) to 100% (complete desiccation, necrosis, or seedling death).

Leaf Relative Water Content (RWC) was determined at 24 and 72 h after spraying to quantify the degree of tissue dehydration induced by the effective dose following the standard turgidity method described by Álvarez-Rodríguez et al. [12]. Fresh weight (FW) of treated leaves was immediately recorded. Leaves were then immersed in deionized water at 4 °C for 24 h in the dark to achieve full turgor, wiped dry, and weighed to obtain the turgid weight (TW). Finally, samples were oven-dried at 70 °C for 48 h to determine the dry weight (DW). RWC was calculated using the following equation:

$$RWC(\%)=\left[\frac{FW-DW}{TW-DW}\right]\times 100.$$

### 2.6. In Vivo Chlorophyll a Fluorescence Imaging and Light Response Curves

To non-invasively assess the functional integrity and biophysical state of the photosynthetic apparatus, chlorophyll *a* fluorescence imaging was performed shortly before plant destructive sampling on both control and treated biological replicates (n=5). Two distinct optical protocols were executed sequentially using an Open FluorCam FC 800-O system (Photon Systems Instruments, Brno, Czech Republic) to comprehensively screen dark-adapted and light-adapted photosynthetic variables as outlined by Maxwell and Johnson [26].

#### 2.6.1. Dark-Adapted Quenching Analysis

Prior to the measurement session, plants were dark-adapted for 20 min to ensure the complete oxidation and relaxation of the Photosystem II (PSII) reaction centers. The quenching protocol was initiated by applying a weak, non-actinic measuring LED light pulse to record the minimum basal fluorescence ($F_{0}$), which was monitored independently as a key indicator of structural damage to the light-harvesting complex antennas. Subsequently, a short saturating light flash ($>2000\;{\mu \mathrm{mol}\;\mathrm{photons}\;m}^{-2}{\;s}^{-1}$) was discharged to determine the maximum dark-adapted fluorescence ($F_{m}$). The maximum quantum yield of PSII (QY_max_) was calculated as

$$\frac{F_{v}}{F_{m}}=\frac{F_{m}-F_{0}}{F_{m}}.$$

Following the dark measurements, plants were exposed to continuous actinic light illumination ($250\;{\mu \mathrm{mol}\;\mathrm{photons}\;m}^{-2}{\;s}^{-1}$) for 5 min to drive photosynthetic biochemistry into a steady state. Periodic saturating pulses were applied during this phase to determine the steady-state maximum fluorescence ($F_{m_{Lss}}$) and steady-state target fluorescence ($F_{\mathrm{Lss}}$).

To avoid transient noise and focus exclusively on stable physiological shifts, the steady-state parameters were specifically selected and calculated by the FluorCam software (Photon Systems Instruments, Drásov, Czech Republic; https://www.psi.cz; accessed 2 July 2026): the effective quantum yield of PSII under light conditions (QY, equivalent to ${F_{v}}^{\prime }/{F_{m}}^{\prime }$), the photochemical quenching coefficient (qP) to estimate the fraction of open PSII reaction centers [27], and the non-photochemical quenching (NPQ) to measure thermal energy dissipation, calculated according to the Stern-Volmer equation

$$NPQ=\frac{F_{m}-F_{m_{Lss}}}{F_{m_{Lss}}}.$$

#### 2.6.2. Light-Adapted Electron Transport Rate (ETR) Curves

Immediately following the steady-state quenching analysis, the electronic transport capacity and plasticity of the seedlings under dynamic light environments were evaluated using the integrated “Light Curve” pre-programmed protocol of the Open FluorCam system. Light adaptation was achieved by exposing the leaves to stepwise, incrementally increasing actinic light intensities (ranging from 0 to $800\;{\mu \mathrm{mol}\;\mathrm{photons}\;m}^{-2}{\;s}^{-1}$). Each light step lasted for 60 s to allow the photosynthetic apparatus to reach a transient equilibrium, concluding with a saturating pulse to calculate the effective quantum yield under that specific light intensity [27].

The Electron Transport Rate (ETR), selected to evaluate the impact of treatments on the whole photosynthetic electron transport chain, was automatically generated for each light level by the FluorCam software using the following allocation formula:

ETR=ΦPSII×PAR×0.5×0.84

where PAR is the photosynthetically active radiation supplied at that specific step, 0.5 is the assumed equal distribution of absorbed photons between PSI and PSII, and 0.84 represents the leaf absorptivity coefficient tailored for standard green leaves. All selected kinetic parameters ($F_{0}$, $F_{v}/F_{m}$, QY, qP, NPQ, and ETR curves), numerical values, and false-color physiological images were processed and extracted using the FluorCam software package.

### 2.7. Spectrophotometric Quantification of Photosynthetic Pigments

To assess whether the biophysical impairments detected by chlorophyll *a* fluorescence were coupled with the chemical degradation of the photosynthetic machinery, the total content of chlorophyll *a* (Chl a), chlorophyll *b* (Chl b), and total carotenoids (Car) was determined quantitatively via spectrophotometric analysis. Fresh leaf tissue samples (50 mg) were collected from the target replicates at 6 and 24 h post-treatment, immediately frozen in liquid nitrogen, and homogenized. The pigment extraction was performed by adding 5 mL of ice-cold 80% (*v*/*v*) acetone. The mixture was kept under continuous agitation in the dark at 4 °C for 24 h to ensure complete extraction.

Following incubation, the homogenates were centrifuged at 4000× *g* for 10 min at 4 °C. The supernatant was collected, and the optical absorbance was measured against an 80% acetone blank at 646.8 nm, 663.2 nm, and 470.0 nm using a UV-Vis double-beam spectrophotometer (Thermo Fisher Scientific, Waltham, MA, USA). The concentrations of individual pigment fractions were calculated using the standardised empirical equations developed by Wellburn [28]. The final values were normalised to the fresh weight of the leaf tissue and expressed as mg g^−1^ Fresh Weight (FW).

### 2.8. Membrane Integrity Degradation Assays

#### 2.8.1. Relative Electrolyte Leakage (EL)

Cell membrane destabilization was assessed via Relative Electrolyte Leakage (EL) at 6 and 24 h post-treatment on tissue samples collected right after the fluorescence measurements. Calibrated leaf discs (1 cm diameter) were excised from the plants, washed briefly with deionized water to remove surface solutes, and incubated in tubes containing 10 mL of deionized water under continuous shaking at room temperature according to the standard method validated by Lutts et al. [29]. The initial electrical conductivity (${\mathrm{EC}}_{1}$) of the solution was measured after 4 h using a benchtop conductivity meter. Samples were then autoclaved at 121 °C for 20 min to release all cellular ions. After cooling down to room temperature, the final electrical conductivity (${\mathrm{EC}}_{2}$) was recorded. The percentage of EL was calculated as $EL(\%)=\left({EC}_{1}/{EC}_{2}\right)\times 100$.

#### 2.8.2. Lipid Peroxidation (MDA Content)

To investigate if membrane disruption was driven by oxidative degradation of lipids, lipid peroxidation was quantified at 24 h by measuring malondialdehyde (MDA) content via the assay described by Hodges et al. [30]. Briefly, frozen leaf tissue (100 mg) was homogenized in 0.1% (*w*/*v*) trichloroacetic acid (TCA). The supernatant obtained after centrifugation was mixed with 0.5% thiobarbituric acid (TBA) in 20% TCA. The mixture was heated at 95 °C for 30 min, quickly cooled in an ice bath, and centrifuged. Absorbance of the supernatant was measured at 532 nm and corrected for non-specific turbidity at 600 nm. MDA concentration was calculated using an extinction coefficient of $155{\;\mathrm{mM}}^{-1}{\;\mathrm{cm}}^{-1}$ and expressed as ${\mathrm{nmol}\;g}^{-1}$ Fresh Weight (FW).

### 2.9. Untargeted GC-MS Metabolomics of Primary Metabolism

To map the metabolic shifts induced by the effective phytotoxic dose of Santolina EO, leaf tissues were collected at 6 and 24 h post-treatment, immediately snap-frozen in liquid nitrogen, and stored at −80 °C.

#### 2.9.1. Metabolite Extraction and Derivatization

Polar metabolites were extracted using a modified methanol/chloroform/water protocol optimized for simultaneous plant primary metabolite analysis [31]. Frozen leaf tissue (50 mg) was ground to a fine powder and extracted with ice-cold 100% methanol containing ribitol ($0.2{\;\mathrm{mg}\;\mathrm{mL}}^{-1}$ in water) as an internal standard. Samples were incubated at 70 °C under agitation, mixed with chloroform, and phase-separated by adding ultrapure water. The upper aqueous-methanol phase containing polar primary metabolites was collected and dried under vacuum in a centrifugal concentrator.

For two-step derivatization, the dried residues were methoximated by adding methoxyamine hydrochloride ($20{\;\mathrm{mg}\;\mathrm{mL}}^{-1}$ in pyridine) and incubating at 37 °C for 90 min under continuous shaking. Trimethylsilylation was subsequently performed by adding *N*-methyl-*N*-(trimethylsilyl)trifluoroacetamide (MSTFA) and incubating at 37 °C for 30 min following standard gas-chromatographic preparation guidelines [32].

#### 2.9.2. GC-MS Analysis and Compound Identification

Derivatized samples (1 μL) were injected in splitless mode into a Gas Chromatography-Mass Spectrometry system equipped with an automated sampler and a 5 MS capillary column (30 m × 0.25 mm × 0.25 μm). Helium was used as the carrier gas at a constant flow rate of 1 mL min^−1^. The injector temperature was set at 250 °C. The GC oven temperature program started at 70 °C (2 min hold), ramped at 5 °C min^−1^ to 320 °C, and was held for 5 min at the final temperature. Mass spectra were acquired in electron ionization (EI) mode at 70 eV with a scanning range of *m*/*z* 50–600. Raw data files were processed for peak deconvolution and alignment. Metabolites (sugars, amino acids, organic acids) were identified by comparing their retention indices (RI) and mass spectral fragmentation patterns against authentic standards and reference open-source database archives, specifically utilising the Golm Metabolome Database [33], MassBank of North America, etc. Peak areas were normalized to the internal standard (ribitol) and leaf fresh weight.

### 2.10. Statistical Analysis

All physiological, phenotypic, and biochemical data, including the spectrophotometric pigment concentrations, were subjected to a one-way Analysis of Variance (ANOVA) followed by Tukey’s Honestly Significant Difference (HSD) post hoc test (α=0.05) to evaluate significant differences among treatment means using statistical software. The non-linear four-parameter log-logistic model for the preliminary screening was evaluated using $R^{2}$ coefficients and standard errors of the estimated parameters. For the untargeted metabolomics dataset, normalised peak intensities were log_10_-transformed and Pareto-scaled. Multivariate statistical analyses, including Principal Component Analysis (PCA) and Partial Least Squares-Discriminant Analysis (PLS-DA), were carried out to identify discriminating metabolites between controls and treated groups. Metabolites exhibiting a Variable Importance in Projection (VIP) score ≥ 1.0 and a *p*-value ≤ 0.05 were considered significant biomarkers of the EO’s herbicidal mode of action.

Metabolite networks were reconstructed separately at 6 and 24 h using Debiased Sparse Partial Correlation (DSPC) analysis. Nodes represented putatively annotated metabolites, whereas edges indicated positive or negative partial correlations. Network topology was evaluated through node degree and betweenness centrality. To simplify network visualization and retain the most relevant metabolites, only nodes with betweenness centrality ≥ 1 and their associated edges were included in the final networks.

## 3. Results

### 3.1. Essential Oils Chemical Characterization

**Figure 1:**
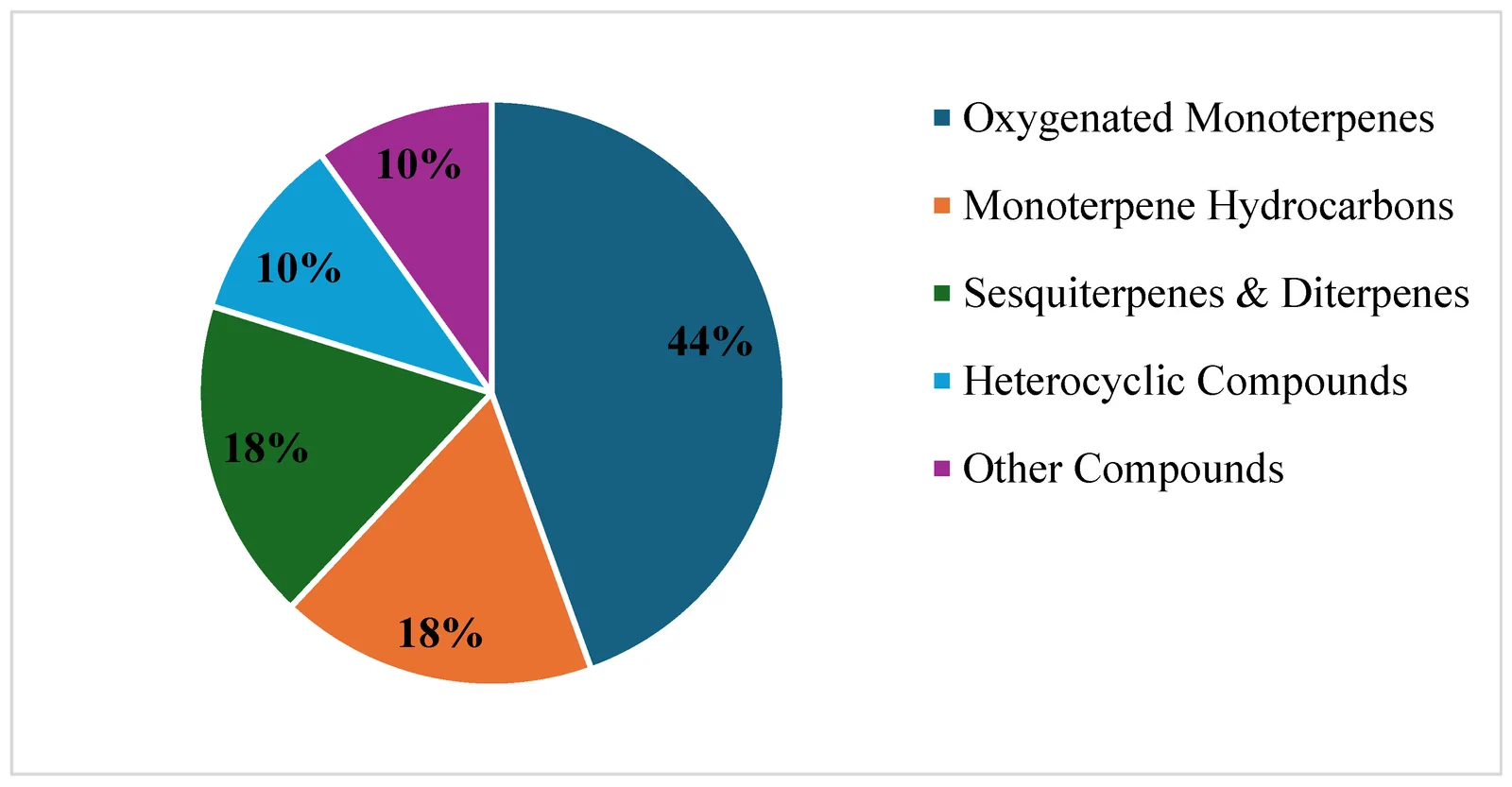
Percentage distribution of the main chemical classes identified in the Santolina essential oil. Values represent uncorrected relative chromatographic peak-area percentages grouped according to chemical class. Because compound-specific response factors were not applied, these distributions describe relative chromatographic abundance and should not be interpreted as absolute mass composition of the EO.

The volatile profile of Santolina essential oil exhibits a high degree of chemical complexity, characterized by the identification of over 100 individual metabolites. The overall composition is predominantly dominated by monoterpene derivatives, with oxygenated monoterpenes constituting the most abundant structural class, closely followed by relatively equal proportions of monoterpene hydrocarbons and sesquiterpene/diterpene fractions (Table 1; Figure 1).

Among the oxygenated monoterpenes, cuminic alcohol showed the highest relative chromatographic abundance, representing 7.09% of the total integrated peak area. This class is further characterised by substantial contributions from cis-carveol (6.10%), cis-pinocarveol (5.86%), and isopinocamphone (4.32%), along with lower amounts of piperitone oxide (1.23%), p-menth-1,4-dien-7-ol (1.43%), and p-mentha-1,5-dien-8-ol (1.09%). Additionally, the presence of artemisia alcohol (1.13%) and artemisia ketone (1.04%), although not the absolute major components, serves as a highly characteristic phytochemical marker of the genus Santolina.

The hydrocarbon monoterpene fraction is primarily driven α-phellandrene and limonene, which showed relative peak-area abundances of 6.15% and 5.87%, respectively. Other low-boiling volatiles, including the genus-specific marker santolinatriene and α-pinene, elute early in the distillation profile but remain restricted to trace or minor quantities.

The heavier terpenoid fraction consists of an intricate mix of sesquiterpenes and diterpenes. Within this group, the sesquiterpene hydrocarbon α-amorphene (4.18%) and the oxygenated sesquiterpene cis-β-elemenone (4.03%) emerge as major constituents. These are accompanied by intermediate levels of α-cedrene epoxide (2.20%), epi-bicyclosesquiphellandrene (1.47%), gamma-gurjunene (1.03%), and the diterpene alcohol cladiell-11-en-7-ol (2.23%).

Interestingly, the analytical profile reveals a distinct presence of heterocyclic nitrogen-containing compounds, which are relatively uncommon in standard essential oil matrices. Specifically, tetramethylpyrazine represents a major component at 5.23%, complemented by an appreciable amount of 3-alkylpyridine (2.10%). The remainder of the essential oil consists of various aliphatic linear molecules, including the long-chain alcohol 2-Z-tridecenol (3.00%), hexyl 2-methylbutanoate (1.14%), and minor organic acids or esters, all contributing to the extract’s highly diverse, multi-component nature.

### 3.2. Preliminary Dose–Response Screening and ED_50_ Estimation

**Figure 2:**
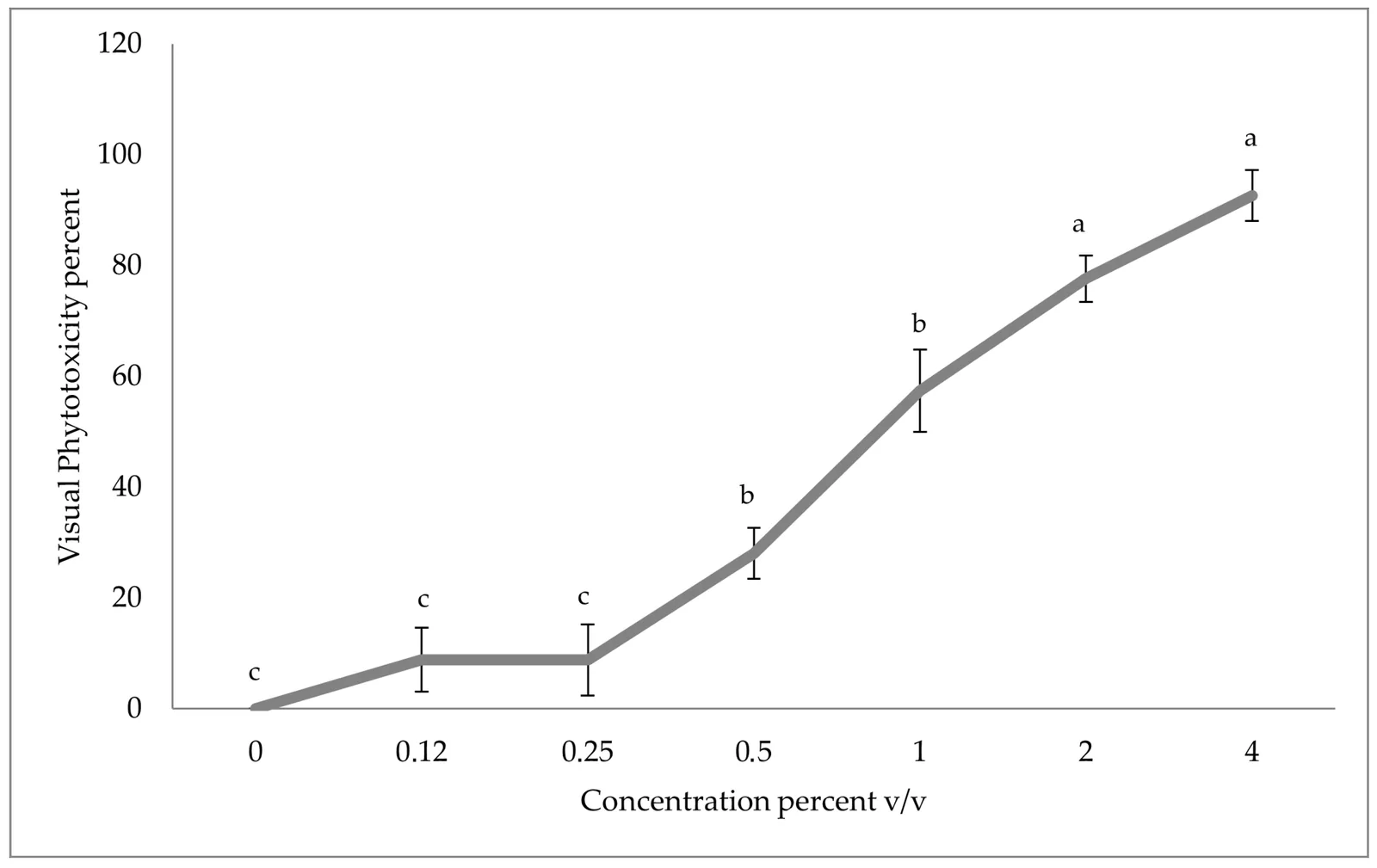
Preliminary post-emergence dose–response screening of *S. chamaecyparissus* (EO) emulsive solution on B. pilosa seedlings at 24 h post-treatment. Visual phytotoxicity is expressed as an injury percentage index ranging from 0% (healthy plant) to 100% (complete leaf desiccation and necrosis). Data points represent experimental means of five independent biological replicates (n=5), while vertical error bars indicate the standard deviation (±SD). Different lowercase letters (a–c) above the markers denote statistically significant differences among concentrations according to one-way Analysis of Variance (ANOVA) followed by Tukey’s Honestly Significant Difference (HSD) post hoc test (α=0.05). The inflection point corresponds to the mathematically calculated ${ED}_{50}$ value (0.82% v/v).

The post-emergence application of *S. chamaecyparissus* essential oil (EO) induced a distinct, concentration-dependent phytotoxic response on *B. pilosa* seedlings at 24 h post-treatment (Figure 2). Visual injury symptoms were completely absent in the negative control (0.0% *v*/*v*). Low concentrations of the EO (0.125% and 0.250% *v*/*v*) resulted in mild, statistically negligible phenotypic damage, yielding mean injury scores of 8.88% ± 5.76% and 8.82% ± 6.43%, respectively.

A sharp inflexion point in phytotoxicity was triggered at 0.500% *v*/*v*, where visible leaf chlorosis and marginal wilting reached 28.06% ± 4.56%. Severe macroscopic desiccation evolved rapidly at 1.000% *v*/*v* (57.38% ± 7.40%) and 2.000% *v*/*v* (77.64% ± 4.17%), culminating in near-complete seedling collapse and extensive necrosis at the highest tested concentration of 4.000% *v*/*v* (92.60% ± 4.63%). Mathematical modelling through the four-parameter log-logistic regression successfully identified the ED_50_, defined as the EO concentration producing a 50% visual injury at 0.82% (*v*/*v*). This effective dose was systematically adopted for all subsequent physiological and biochemical evaluations.

### 3.3. Phenotypic Injury Progression and Leaf Hydration Dynamics

**Figure 3:**
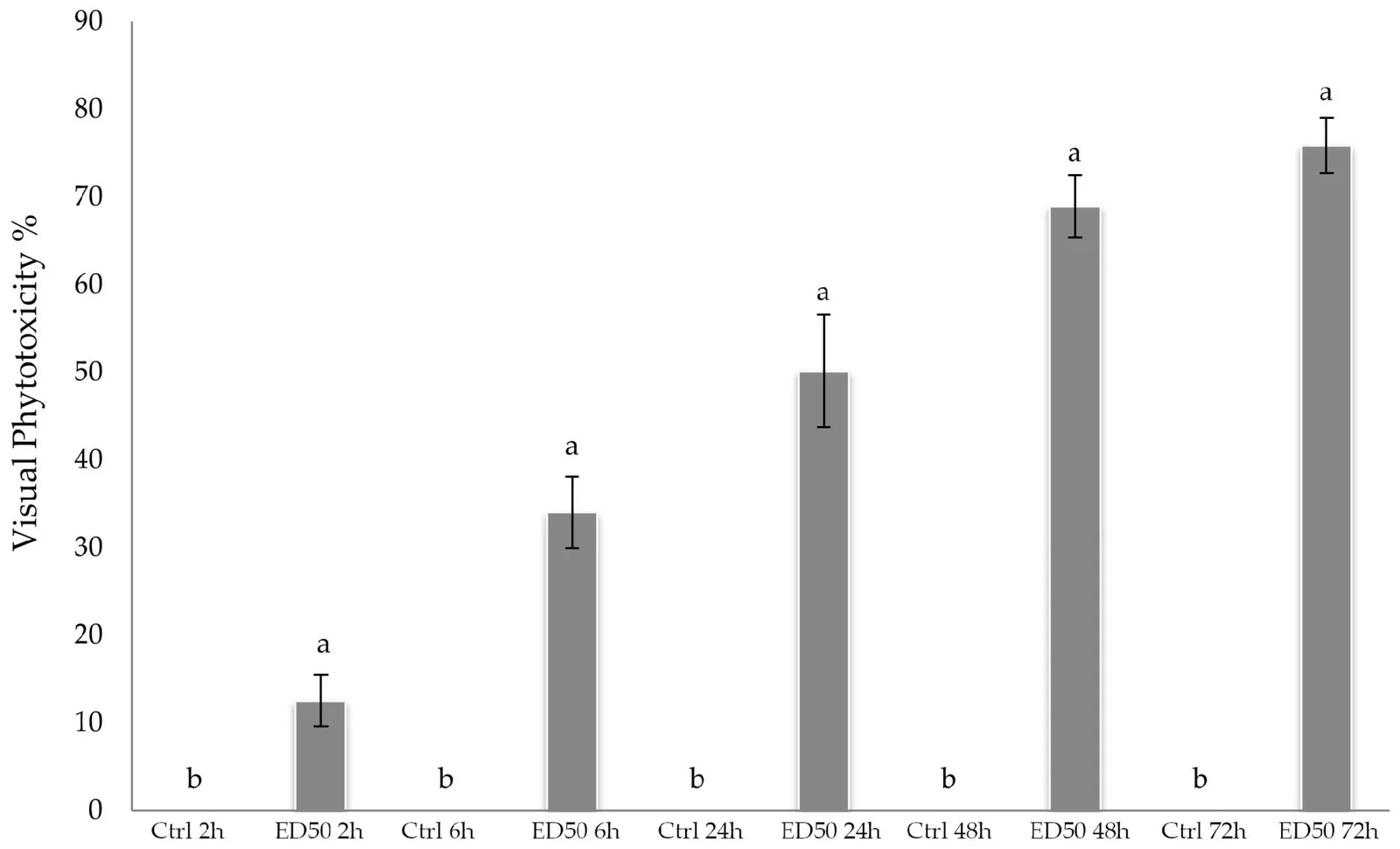
Kinetic progression of visual phytotoxicity induced by the effective ${ED}_{50}$ concentration (0.82% v/v) of *S. chamaecyparissus* (EO) emulsified solution on *B. pilosa* L. seedlings over a 72 h timeline. Bars represent the means of five independent biological replicates (n=5), categorized by time points from left to right: 2, 6, 24, 48, and 72 h post-application. Vertical error bars indicate the standard deviation (±SD). Different lowercase letters (a, b) within each time point pair denote statistically significant differences between the negative control (Ctrl) and the treated cohort (EOT) according to one-way Analysis of Variance (ANOVA) followed by Tukey’s Honestly Significant Difference (HSD) post hoc test (α=0.05).

The kinetic monitoring of *B. pilosa* seedlings treated with the fixed ${ED}_{50}$ dose highlighted a rapid “burning” herbicidal effect. Initial symptoms of phytotoxicity were visibly apparent as early as 2 h post-treatment, showing a mean score of 12.52% ± 2.94%. The injury progression curve advanced linearly to 34.02% ± 4.09% at 6 h, stabilizing around the predicted mid-point value of 50.14% ± 6.43% at 24 h. Prolonged exposure led to widespread, irreversible laminar desiccation, with visual injuries reaching 68.92% ± 3.54% at 48 h and maximizing at 75.86% ± 3.16% at 72 h. Throughout the 72 h timeline, negative control plants maintained full phenotypic vigor (0.00% ± 0.00% injury) (Figure 3).

This macroscopic desiccation correlated tightly with a severe drop in leaf hydration status. Control plants maintained a stable and optimal Relative Water Content (RWC), oscillating between 87.84% ± 1.22% (24 h) and 89.08% ± 1.21% (72 h). Conversely, the formulation applied at the ED50 level triggered an acute water deficit. Leaf RWC significantly collapsed to 62.58% ± 6.00% within 24 h and further deteriorated to a critical value of 39.96% ± 4.22% at 72 h, demonstrating an absolute failure of cellular water retention capacity (Figure 3).

### 3.4. Photosynthetic Pigments Destruction Kinetics

**Figure 4:**
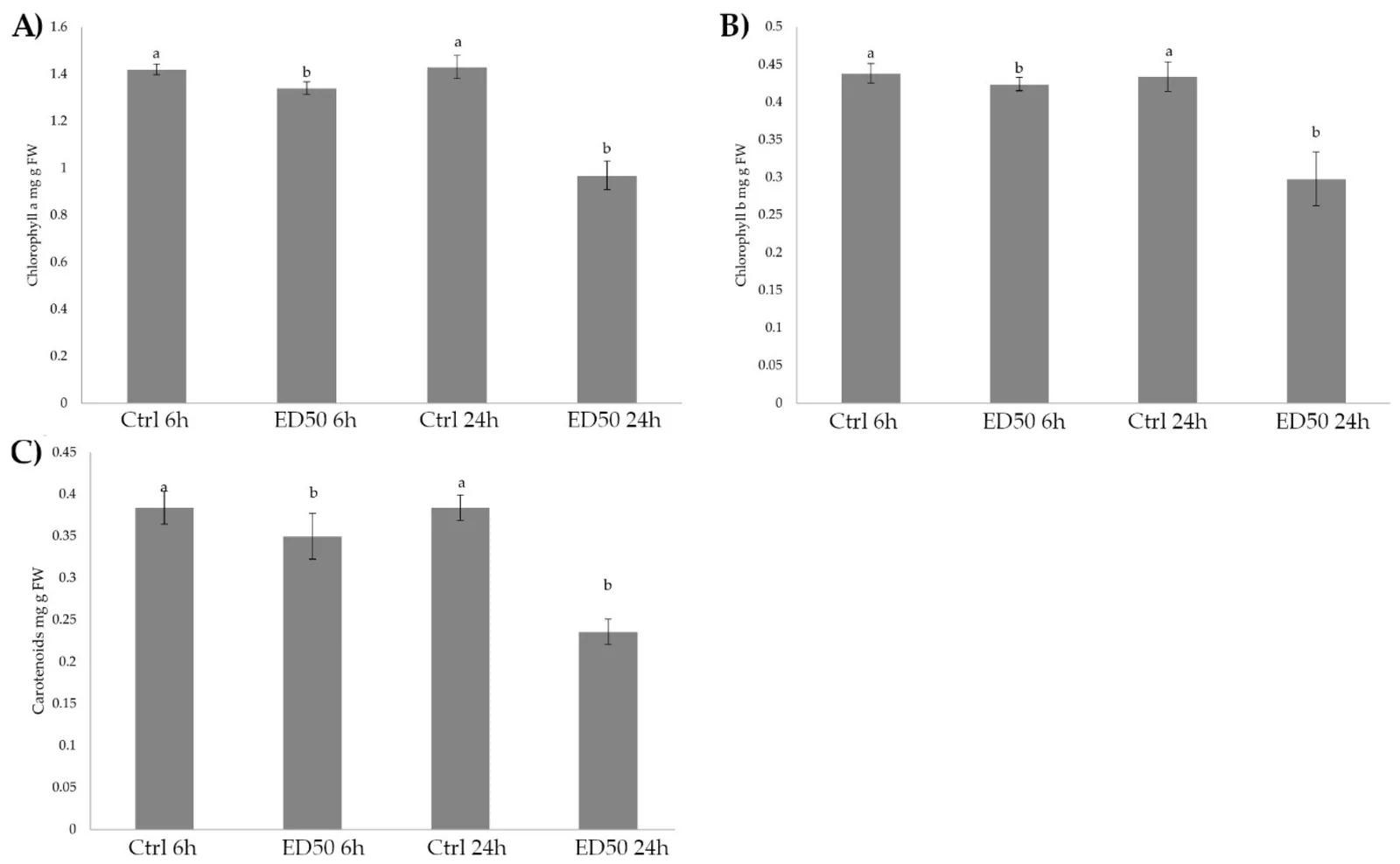
Dynamics of photosynthetic pigment contents in *B. pilosa* leaves at 6 and 24 h post-treatment with the *S. chamaecyparissus* EO at the ED_50_ concentration (0.82% v/v). (**A**) Chlorophyll *a* (Chl a), (**B**) Chlorophyll *b* (Chl b), and (**C**) Total Carotenoids (Car). Bars represent the experimental means of five independent biological replicates (n=5), and vertical error bars indicate the standard deviation (±SD). Different lowercase letters above the bars denote statistically significant differences among treatment groups within each individual sampling time point according to one-way Analysis of Variance (ANOVA) followed by Tukey’s Honestly Significant Difference (HSD) post hoc test (α=0.05). Pigment concentrations are expressed as ${mg\;g}^{-1}$ of leaf Fresh Weight (FW).

Spectrophotometric quantification revealed that the immediate structural damage to the photosynthetic apparatus was not driven by the sudden chemical breakdown of light-harvesting pigments. At 6 h post-treatment, despite the early biophysical disturbances typical of the EO action, the leaf content of Chlorophyll *a* (Chl a), Chlorophyll *b* (Chl b), and Total Carotenoids (Car) in the EOT cohort remained statistically comparable to the untreated control groups. Specifically, at 6 h, Chl a contents were 1.34 mg g^−1^ FW in treated leaves versus 1.42 mg g^−1^ FW in the control; similarly, Chl b (0.42 vs 0.44 mg g^−1^ FW) and Car (0.35 vs 0.38 mg g^−1^ FW) showed no significant early degradation (Figure 4A–C).

A massive, statistically significant degradation of the entire pigment pool was established tardily at 24 h. In treated seedlings, Chl a levels plummeted by approximately 32.5%, shifting down to 0.97 mg g^−1^ FW compared to the stable baseline of 1.43 mg g^−1^ FW observed in the control (Figure 4A). Parallel destructive patterns were observed for both Chl b (dropping to 0.30 mg g^−1^ FW) (Figure 4B) and Total Carotenoids (decreasing to 0.24 mg g^−1^ FW) (Figure 4C).

### 3.5. Membrane Integrity Degradation and Lipid Peroxidation

**Figure 5:**
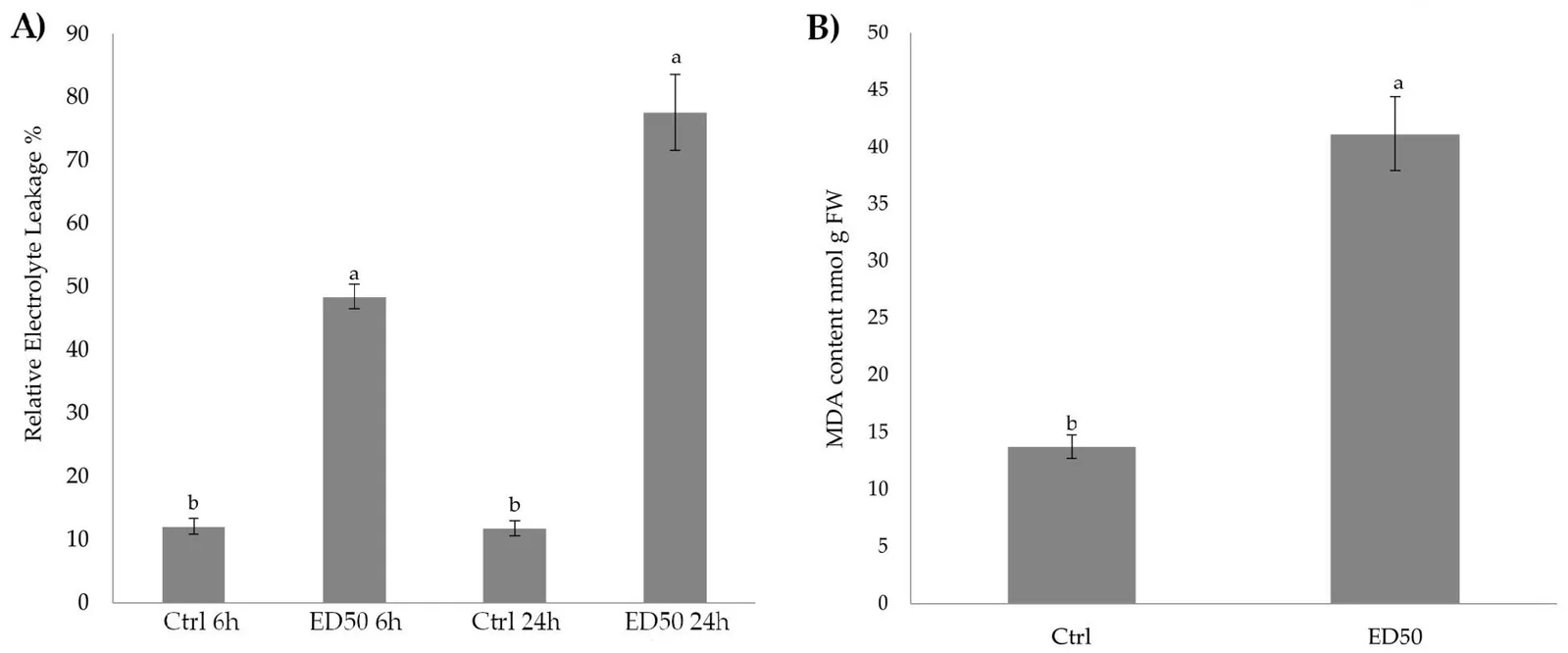
Impact of *S. chamaecyparissus* (EO) applied at ED_50_, (0.82% v/v) on cell membrane stability of *B. pilosa* leaves. (**A**) Relative Electrolyte Leakage (EL) monitored at 6 and 24 h post-treatment. (**B**) Malondialdehyde (MDA) content quantified at 24 h post-treatment as a biomarker of lipid peroxidation. Bars represent the experimental means of five independent biological replicates (n=5), and vertical error bars indicate the standard deviation (±SD). Different lowercase letters above the bars denote statistically significant differences between treatments according to one-way Analysis of Variance (ANOVA) followed by Tukey’s Honestly Significant Difference (HSD) post hoc test (*p* ≤ 0.05). Values are expressed as percentages (%) for EL and nanomoles per gram of fresh weight (${nmol\;g}^{-1}\;FW$) for MDA.

The primary mode of action of *S. chamaecyparissus* EO was strictly validated by an early, massive disruption of cellular membrane permselectivity. Untreated control leaves exhibited negligible and constant baseline values of Relative Electrolyte Leakage (EL), centered around 12.10% ± 1.26% (6 h) and 11.76% ± 1.21% (24 h). In contrast, foliar application of the ${EC}_{50}$ solution induced immediate membrane permeabilization, causing cellular ion leakage to jump up to 48.38% ± 1.96% within just 6 h. This destructive trend escalated drastically at 24 h, where treated tissues reached a severe solute leakage value of 77.58% ± 6.05%, indicating generalized cellular lysis (Figure 5A).

To determine whether this structural membrane breakdown was driven by oxidative stress, the lipid peroxidation byproduct was quantified at 24 h. Seedlings exposed to the effective dose of the essential oil exhibited an acute accumulation of malondialdehyde (MDA). The MDA content surged significantly to 41.16 ± 3.26 nmol g^−1^ FW, representing a distinct three-fold increase relative to the basal levels found in healthy control tissues (13.72 ± 1.03 nmol g^−1^ FW) (Figure 5B). This biochemical fingerprint confirms that the EO-induced membrane disruption is tightly coupled with extensive oxidative damage to the lipid bilayers.

### 3.6. In Vivo Chlorophyll a Fluorescence Biophysical Dynamics

**Figure 6:**
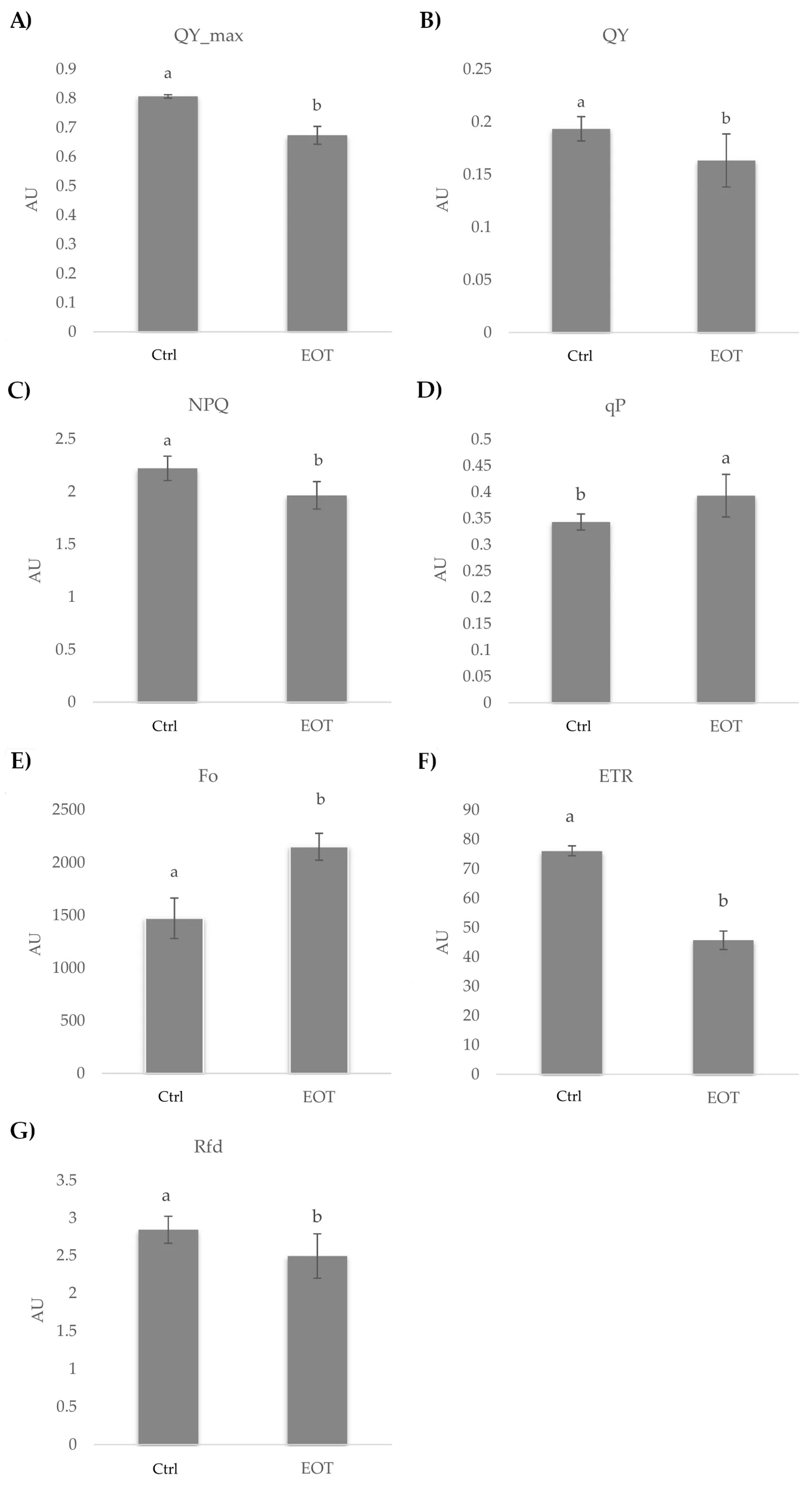
Biophysical variations in dark-adapted and light-adapted chlorophyll *a* fluorescence parameters (QY_max, QY, NPQ, qP, $F_{0}$, ETR, and Rfd) of *B. pilosa* leaves subjected to the ED_50_ *S. chamaecyparissus* (EO). (**A**) maximum quantum yield of PSII in dark-adapted leaves (QY_max_, equivalent to F_v_/F_m_); (**B**) effective quantum yield of PSII under light-adapted conditions (QY); (**C**) non-photochemical quenching (NPQ); (**D**) photochemical quenching coefficient (qP); (**E**) minimum fluorescence (F_0_); (**F**) apparent electron transport rate (ETR); and (**G**) fluorescence decline ratio (Rfd). Columns represent the experimental means of independent biological replicates (n=5), while vertical error bars indicate the standard deviation (±SD). Different lowercase letters (a, b) above the bars denote statistically significant differences between the Ctrl and EOT groups according to one-way Analysis of Variance (ANOVA) followed by Tukey’s Honestly Significant Difference (HSD) post hoc test (*p* ≤ 0.05). Values on the *Y*-axis are expressed in arbitrary units (AU).

The evaluation of *B. pilosa* photosynthetic functionality through in vivo chlorophyll a fluorescence imaging revealed extensive biophysical impairments induced by the *S. chamaecyparissus* essential oil treatment. In dark-adapted leaves, the maximum quantum yield of Photosystem II (QY_max_, equivalent to F_v_/F_m_) exhibited a statistically significant decrease, dropping from an optimal physiological baseline of 0.81 ± 0.01 in the control plants to 0.67 ± 0.03 in the treated cohort (*p* < 0.05) (Figure 6A). This restriction in vertical maximum energy conversion efficiency was tightly synchronized with a major elevation in the minimum basal fluorescence (F_0_), which surged from 1471.8 ± 192.1 a.u. in the control leaves to a significantly higher value of 2149.2 ± 126.0 a.u. in the treated plants (Figure 6E). This severe inflation of F_0_ provides solid biophysical evidence of the structural dissociation or physical damage within the light-harvesting complex (LHCII) antenna proteins.

Under continuous actinic light exposure, steady-state photosynthesis showed an altered distribution of excitation energy. The effective quantum yield of Photosystem II (QY) significantly decayed from 0.19 ± 0.01 (Ctrl) to 0.16 ± 0.03 (EOT) (Figure 6B). Interestingly, the photochemical quenching coefficient (qP) showed an unexpected, statistically significant upward shift in the treated group (0.39 ± 0.04, denoted by letter a) compared to the healthy control cohort (0.34 ± 0.02, denoted by letter b) (Figure 6D). Conversely, the non-photochemical dissipation channels were severely compromised, as shown by a significant decrease in the steady-state non-photochemical quenching (NPQ), which dropped from 2.22 ± 0.12 in controls to 1.96 ± 0.13 in treated individuals (Figure 6C).

Consistent with the decline in PSII operating efficiency, the apparent electron transport rate (ETR) decreased significantly from 76.1 ± 1.7 in control leaves to 45.7 ± 3.1 in EOT leaves (Figure 6F). The fluorescence decline ratio (Rfd), an integrative indicator of photosynthetic vitality, was likewise significantly reduced from 2.8 ± 0.18 in the control group to 2.5 ± 0.29 following EO treatment (Figure 6G). Together, these changes indicate that the EO treatment restricted photosynthetic electron flow and reduced the overall functional performance of the photosynthetic apparatus.

### 3.7. Untargeted Metabolomic Analysis

**Figure 7:**
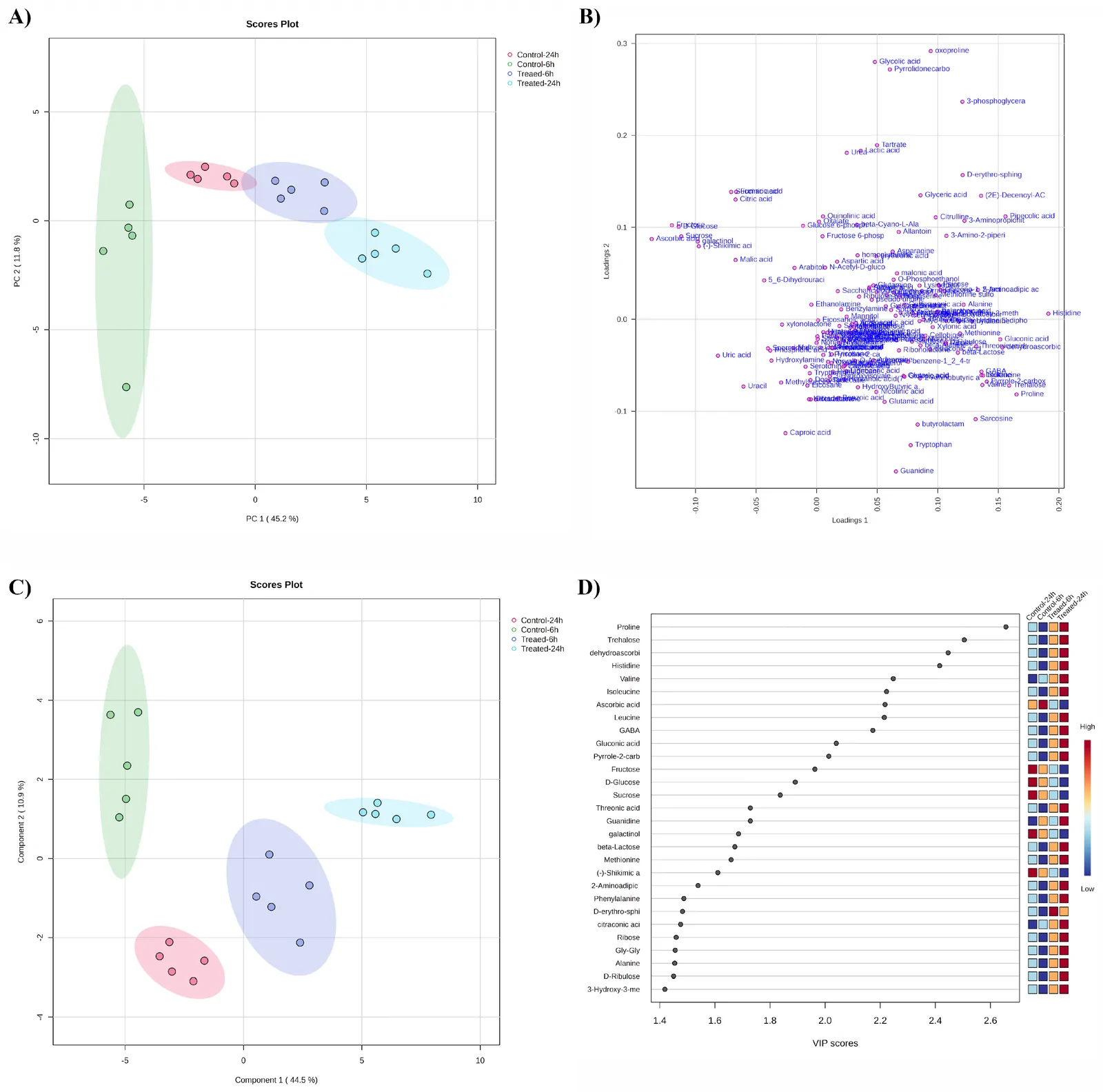
Multivariate statistical analysis of the untargeted GC-MS metabolic profiles of *B. pilosa* leaves monitored at 6 and 24 h post-treatment with the *S. chamaecyparissus* (EO) essential oil at the ED_50_ concentration (0.82% v/v) (**A**). Unsupervised Principal Component Analysis (PCA) score plot showcasing the global sample clustering and kinetic distribution (**B**). PCA loading plot illustrating the distribution of the individual putatively annotated metabolites across the multivariate space (**C**). Supervised Partial Least Squares-Discriminant Analysis (PLS-DA) score plot maximizing class discrimination among the experimental groups (**D**). Variable Importance in Projection (VIP) plot displaying the top discriminant biomarkers with a score threshold > 1.4; the corresponding mini-heatmap on the right indicates the relative abundance of each metabolite across the four experimental groups (Control-6 h, Control-24 h, Treated-6 h, and Treated-24 h), color-coded from dark blue (low) to dark red (high). In both score plots, the shaded ellipses define the 95% confidence intervals for each individual treatment cohort based on five independent biological replicates (n=5).

The unsupervised multivariate analysis performed via Principal Component Analysis (PCA) clearly distinguished the global metabolic alterations induced by the *S. chamaecyparissus* essential oil on *B. pilosa* leaves, explaining a cumulative 57% of the total variance across the first two principal components. Along the primary axis (PC1, 45.2%), a robust treatment-dependent separation is visible, positioning the untreated control cohorts (Control-6 h and Control-24 h) entirely within the negative quadrants, whereas the ${ED}_{50}$ herbicidal application shifts the metabolic profiles toward the positive right quadrants. The secondary axis (PC2, 11.8%) effectively captures the kinetic progression of the phytotoxic stress, showing a marked diagonal trajectory from the intermediate Treated-6 h cluster to the highly altered Treated-24 h group, which isolates itself at the furthest extremity of the multivariate space (Figure 7).

To validate the analytical accuracy and reproducibility of the GC-MS platform, a separate PCA including the Quality Control (QC) samples was performed as a methodological check and is provided in the Supplementary Table S1. In this supplementary model, all biological replicates maintain the exact same treatment-dependent clustering pattern, while the injected QC replicates gather tightly together in a central, symmetric position within the space, confirming the absolute stability of the chromatographic system and the absence of significant analytical drift throughout the experimental sequence (Figure 7).

The corresponding loading plot from the main analysis identifies the specific biochemical drivers behind this spatial clustering. The negative side of PC1, associated with healthy control plants, is strongly characterized by high levels of primary soluble sugars and vital antioxidants, including fructose, D-glucose, sucrose, galactinol, and ascorbic acid, as well as the shikimic acid pathway precursor. Conversely, the positive side of PC1 reflects the treated plants’ severe physiological distress, driven by the massive accumulation of cellular stress biomarkers and free amino acids, most notably proline, valine, isoleucine, threonine, GABA, and trehalose. Finally, the vertical separation along PC2 is largely driven by metabolites like oxoproline, glycolic acid, pyrrolidonecarboxylic acid, and 3-phosphoglycerate, which act as early kinetic markers of the immediate photorespiratory and bioenergetic stress response initiated within the first 6 h of chemical treatment (Figure 7).

To maximise class separation and isolate the most statistically robust biomarkers responsible for the observed phytotoxic alterations, the exploratory unsupervised data were further processed using a supervised Partial Least Squares-Discriminant Analysis (PLS-DA). The resulting PLS-DA score plot revealed a clear separation among the four experimental groups, with Component 1 (44.5%) and Component 2 (10.9%) accounting for 55.4% of the total sample covariance. The spatial arrangement strongly mirrored the PCA trajectories, maximizing the distance between the untreated control cohorts on the left side of the plot and the distinct kinetic stages of the EOT group on the right (Figure 7).

The statistical reliability and predictive power of this discriminant model were rigorously confirmed by cross-validation and permutation parameters. The cross-validation analysis identified that the model achieves optimal performance using three latent components, yielding a high accuracy, an $R^{2}$ value close to 0.97, and a robust cross-validated predictability index ($Q^{2}$) exceeding 0.85, marked as the optimal classifier. Furthermore, the high predictive validity of the model was verified by a 20-fold permutation test, in which the observed statistic was isolated at the extreme right edge of the distribution (p<0.05, 0/20), successfully ruling out any overfitting or random class assignment (Figure 7).

The Variable Importance in Projection (VIP) scores were evaluated to identify the key metabolic drivers of this group discrimination, selecting the top biomarkers with VIP values > 1.4. In perfect agreement with the observed physiological distress, proline and trehalose emerged as the most prominent discriminant metabolites, showing markedly higher and exclusive abundance in the Treated-24 h cohort. The VIP plot also captured the sharp accumulation of free amino acids such as histidine, valine, isoleucine, and leucine, alongside GABA and gluconic acid, as signature responses of the late-stage phytotoxic stress. Conversely, key structural sugars and antioxidant defences, including dehydroascorbic acid, ascorbic acid, fructose, D-glucose, sucrose, and galactinol, displayed their highest levels in the control groups and a severe decline following the herbicidal oil exposure, further validating the total disruption of the plant’s bioenergetic and redox homeostasis (Figure 7).

The hierarchical clustering heatmap of all statistically significant metabolites identified by ANOVA reveals a distinct distribution pattern across the four experimental groups. The dendrogram clusters the samples into two main branches, isolating the Treated-24 h group from a macro-cluster containing the Control-6 h, Control-24 h, and Treated-6 h groups. Regarding the individual molecular modulations, a major cluster encompassing amino acids and alternative carbohydrates, such as ornithine, alanine, glycine, phenylalanine, histidine, lysine, proline, valine, leucine, GABA, and trehalose, shows a strong, synchronized up-regulation exclusively in the Treated-24 h group, whereas these features maintain low relative abundances across both control points and intermediate levels at Treated-6 h. A separate, specific group of compounds, including citric acid, succinic acid, fumaric acid, nicotianamine, and cellobiose, exhibits a transient, high accumulation restricted to the Treated-6 h group, followed by a substantial decline in the Treated-24 h cohort. Conversely, a clear down-regulation trend is observed for a cluster composed of primary soluble sugars and organic acids, namely sucrose, D-glucose, fructose, galactinol, malic acid, ascorbic acid, and (−)-shikimic acid, which maintain their highest relative levels in both Control-6 h and Control-24 h samples, show an initial decrease at Treated-6 h, and reach their minimum relative abundance in the Treated-24 h group. Finally, a small cluster at the lower edge of the map, including uric acid, uracil, and methylsuccinic acid, shows an exclusive, marked abundance in the Control-6 h group compared to all other analyzed conditions.

### 3.8. Debiased Sparse Partial Correlation (DSPC) Network Analysis

**Figure 8:**
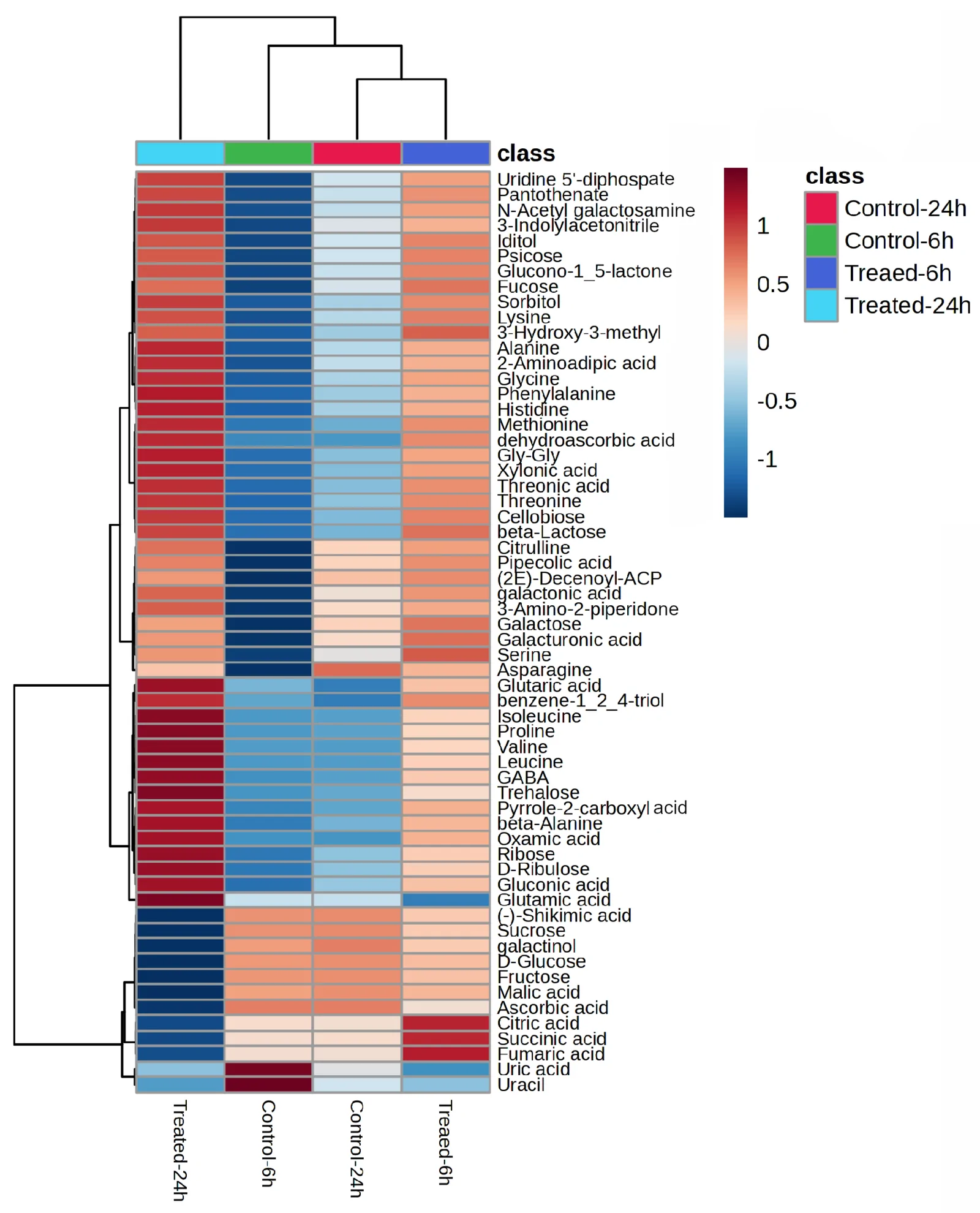
Hierarchical clustering heatmap of all statistically significant metabolites identified by ANOVA in *B. pilosa* leaves monitored at 6 and 24 h post-treatment with the *S. chamaecyparissus* (EO) essential oil at the ED_50_ (0.82% v/v). Both rows (metabolites) and columns (experimental groups) are clustered using Euclidean distance and Ward’s linkage method based on their normalised relative abundances. The top group-classification bar differentiates the four experimental conditions: Control-6 h (green), Control-24 h (red), Treated-6 h (dark blue), and Treated-24 h (light blue). The vertical colour scale on the right represents the standardised relative metabolite levels, ranging from dark blue (low relative abundance, −1) to dark red (high relative abundance, 1). Each column represents the mean values obtained from five independent biological replicates (n=5).

**Figure 9:**
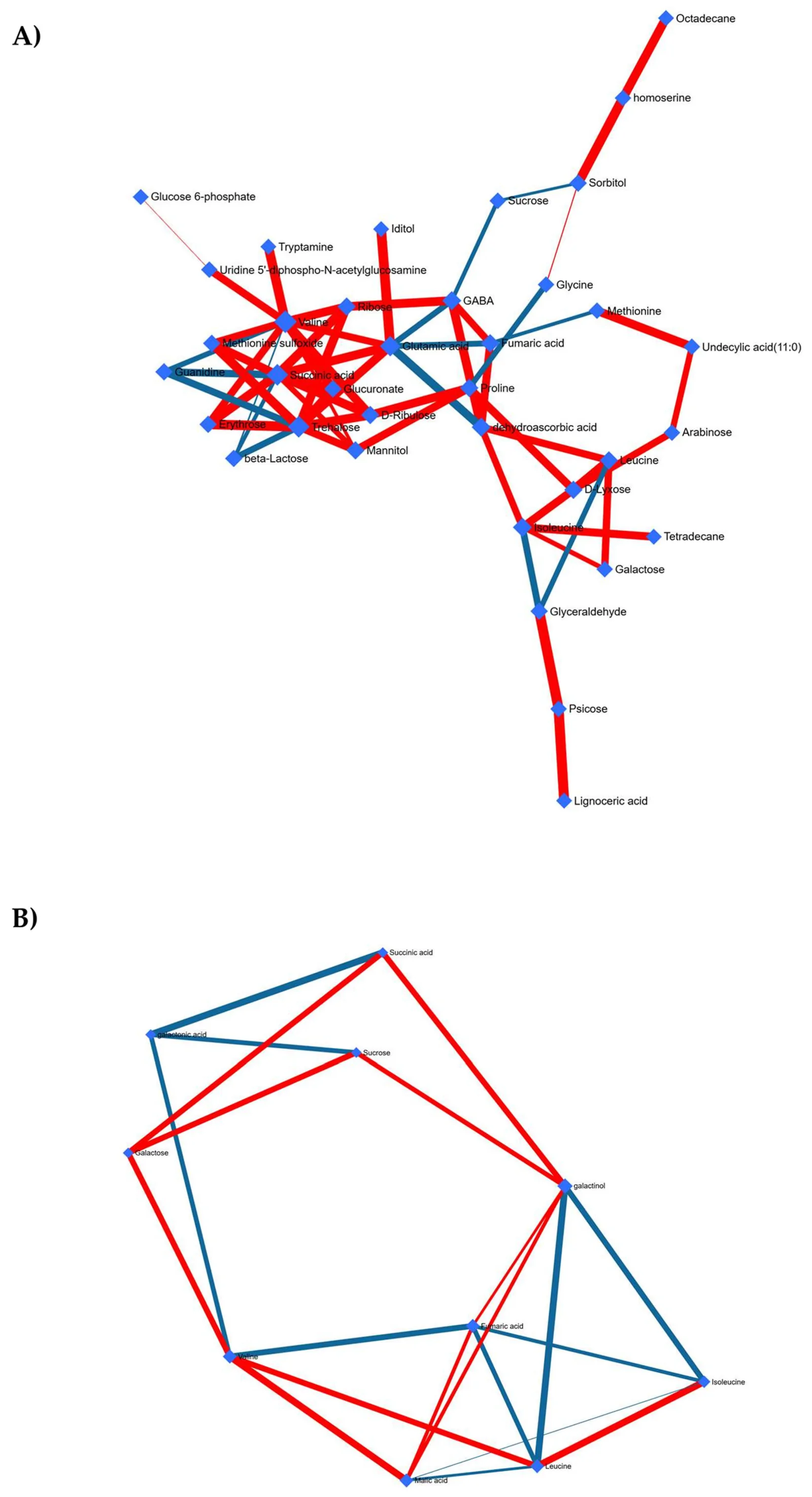
Graphical network analysis based on Debiased Sparse Partial Correlation (DSPC) modeling of the *B. pilosa* leaf metabolome under *S. chamaecyparissus* (EO) stress at distinct kinetic intervals (n=10 samples per network, representing 5 control and 5 treated biological replicates). (**A**) The 6 h post-treatment network configuration showcasing a highly centralized, interconnected topological layout. (**B**) The 24 h post-treatment network model showing a severe structural contraction and pathway fragmentation. Blue diamond nodes represent the individual putatively annotated metabolites, while connecting edges represent significant partial correlation values. Edge color indicates the direction of the correlation (red for positive correlations, blue for negative correlations), and edge thickness is proportional to the strength of the statistical association.

To characterise the structural topological rearrangements and coregulatory shifts in the leaf metabolome of *B. pilosa*, separate Debiased Sparse Partial Correlation (DSPC) network analyses were modelled for the 6 h and 24 h post-treatment experimental intervals (Figure 8 and Figure 9A,B).

The topological properties calculated for the 6 h network revealed a highly dense and centralised graphic configuration, capturing a wide array of coordinated metabolic interactions early after the essential oil application. The 6 h network structure was highly interconnected around two primary topological hubs possessing the highest degree of connectivity (K=11 for valine, and K=9 for both trehalose and succinic acid). These central nodes coordinated a dense core regulatory cluster comprising primary carbon and nitrogen assimilation endpoints, with glutamic acid displaying the highest betweenness centrality score (184.26, K=7), followed by dehydroascorbic acid (145.02, K=5), proline (111.77, K=4), and isoleucine (99.34, K=5). Other key interactive features within this early-stage framework included GABA (K=5), fumaric acid (K=4), leucine (K=4), and various mono- and oligosaccharides such as mannitol, sorbitol, ribose, and sucrose.

In contrast, the topological architecture underwent a severe contraction and fragmentation at the 24 h interval, characterised by a dramatic loss of node abundance and connectivity across the network space. The 24 h network was reduced to a singular, minimalist circular loop consisting of only ten remaining active nodes, indicating a generalised operational de-coregulation. Within this contracted late-stage model, the maximum degree values dropped significantly, with galactinol emerging as the highest connected node (K=6, betweenness centrality = 9.13), followed closely by valine (K=5, betweenness centrality = 7.53), fumaric acid (K=5), leucine (K=5), and malic acid (K=5). The remaining nodes—comprising isoleucine (K=4), galactose (K=3), succinic acid (K=3), sucrose (K=3), and galactonic acid (K=3)—formed a peripheral, restricted structural circuit governed exclusively by positive partial correlation vectors, highlighting the complete disappearance of the central multi-pathway coordination hubs observed at the 6 h interval.

## 4. Discussion

The present study demonstrates that the essential oil obtained from *S. chamaecyparissus* exerts rapid, concentration-dependent post-emergence phytotoxicity against *B. pilosa*. The estimated ED_50_ of 0.82% (*v*/*v*), the visible injury observed within 2 h, and the progressive desiccation observed over 72 h describe a response consistent with that of a contact bioherbicide. Essential oils generally act rapidly on exposed plant surfaces, and their efficacy depends strongly on spray coverage, formulation, and penetration through the cuticle, rather than on extensive systemic translocation [8,34]. Their hydrophobicity and volatility may therefore limit field performance through phase instability, heterogeneous deposition, and rapid evaporative losses. Although the EO emulsions used here were freshly prepared and homogenised immediately before spraying, their physicochemical stability and droplet-size distribution were not formally characterised. Future studies should therefore optimise formulation and application conditions to improve dispersion, leaf retention, and persistence, while assessing emulsion stability, droplet size, spray deposition, and performance under field conditions. The response observed here is also consistent with the marked sensitivity of *B. pilosa* to *Carlina acaulis* essential oil, which caused leaf necrosis, loss of relative water content, and inhibition of PSII activity in established plants [12].

Previous studies also indicate that the phytotoxic activity of *S. chamaecyparissus* EO is strongly dependent on the target weed species. Jouini et al. [7] evaluated this EO against *Avena fatua*, *Echinochloa crus-galli*, *Portulaca oleracea*, and *Amaranthus retroflexus*, reporting dose-dependent activity and marked differences in susceptibility among species, with *A. retroflexus* showing particularly high sensitivity. Likewise, Verdeguer et al. [35] demonstrated pre- and post-emergence phytotoxicity against *Erigeron bonariensis*, although the efficacy of *S. chamaecyparissus* EO was lower than that of some of the other essential oils tested. Together with the present results on *B. pilosa*, these findings suggest that *S. chamaecyparissus* EO possesses herbicidal activity across several weed taxa, but also highlight that its efficacy is strongly species- and application-dependent.

Taken together, these findings further support *S. chamaecyparissus* EO as a source of post-emergence phytotoxic activity while emphasizing the need to establish its spectrum of weed susceptibility and selectivity.

The strong biological response is consistent with the oil’s chemically complex nature. Oxygenated monoterpenes represented the dominant fraction, while monoterpene hydrocarbons and sesquiterpene/diterpene compounds also contributed substantially to the profile. Previous studies have shown pronounced interspecific and chemotypic variability within the genus *Santolina*, with major constituents changing according to species, provenance, phenological stage, and extraction conditions [34,36,37]. Accordingly, the profile reported here should be regarded as the fingerprint of the specific plant material and single EO batch investigated in the present study, rather than as a composition representative of *S. chamaecyparissus* as a species. For the same reason, the phytotoxic, physiological, and metabolic responses described here should be interpreted specifically in relation to this chemically characterized EO batch. Although the use of a single batch ensured internal consistency among the dose–response, physiological, biochemical, fluorescence, and metabolomic experiments, it did not allow batch-to-batch variability in chemical composition or biological activity to be assessed. Future studies should therefore compare independent EO batches obtained from different populations, harvesting periods, phenological stages, and environmental conditions to determine the reproducibility and generalisability of the observed phytotoxic effects. Within the batch investigated here, cuminic alcohol, cis-carveol, cis-pinocarveol, limonene, phellandrene, isopinocamphone, and several oxygenated sesquiterpenes may all contribute to phytotoxicity because lipophilic terpenoids can interact with membranes and other cellular targets [34]. Nevertheless, the present experiment does not allow the effect to be assigned to any single constituent. Additive, synergistic, or antagonistic interactions among major and minor components remain plausible and should be resolved through bioassays with authentic standards and reconstructed mixtures [38,39,40].

The temporal sequence of the measured responses indicates that membrane destabilisation was an early and central event in the phytotoxic process. Relative electrolyte leakage increased sharply within 6 h, whereas chlorophyll *a*, chlorophyll *b*, and carotenoid contents were still statistically unchanged at that time. By 24 h, electrolyte leakage approached 80%, malondialdehyde content had increased approximately threefold, and significant pigment depletion was evident. This ordering suggests that the loss of membrane permeability and selectivity and the oxidative deterioration of membrane lipids preceded the bulk degradation of the photosynthetic pigment pool. Comparable combinations of electrolyte leakage, lipid peroxidation, pigment loss, and tissue necrosis have been reported after exposure to essential oils from *Artemisia fragrans*, *Artemisia argyi*, *Pogostemon benghalensis*, and *Litsea pungens* [9,10,41,42]. The parallel decline in leaf relative water content and increase in visual injury are therefore consistent with an inability of damaged cells to retain ions and water, leading to loss of turgor, laminar desiccation, and irreversible tissue collapse.

Chlorophyll fluorescence analysis showed that functional damage to photosynthesis developed before extensive pigment destruction. The decreases in maximum and effective PSII quantum yields, electron transport rate, and Rfd, together with the increase in Fo, are consistent with impaired excitation-energy transfer, restricted PSII photochemistry, and reduced downstream electron transport [26]. Similar reductions in Fv/Fm and operating PSII efficiency have been observed in *B. pilosa* treated with *C. acaulis* essential oil and in weeds exposed to *A. fragrans* or *A. argyi* oils [9,10,12]. These variables should, however, be interpreted as integrated indicators of photosynthetic injury rather than as proof of a unique molecular target. In particular, the increase in qP should not be considered evidence of improved photochemical performance because it occurred simultaneously with reductions in PSII yield and electron transport. It may instead reflect lower excitation pressure on the remaining active centres due to reduced functional antenna size, heterogeneous tissue necrosis, or diminished light absorption. Likewise, the decline in NPQ indicates that regulated thermal dissipation could not be sustained under the imposed injury. This differs from the increase in regulated non-photochemical dissipation reported in *B. pilosa* exposed to *C. acaulis* oil [12], suggesting that the *Santolina* treatment may have driven the tissue beyond a reversible photoprotective phase.

The metabolomic response supports a transition from an early compensatory phase to widespread metabolic dysfunction. At 6 h, the transient enrichment of citric, succinic, and fumaric acids, along with other organic acids, may reflect a short-lived attempt to maintain respiratory energy production and redox balance during acute chemical stress. By 24 h, however, the marked accumulation of proline, GABA, branched-chain amino acids, and trehalose indicated severe osmotic and oxidative stress. A broadly comparable increase in amino acids, accompanied by depletion of several soluble sugars, was reported in *B. pilosa* after exposure to *C. acaulis* essential oil [12]. The accumulation of valine, leucine, and isoleucine should not be interpreted solely as de novo synthesis of protective osmolytes; in *Arabidopsis thaliana*, osmotic stress can increase branched-chain amino-acid pools through abscisic-acid-regulated protein degradation [43]. Thus, the late amino-acid signature may reflect both an attempted protective response and enhanced proteolysis associated with tissue injury. Trehalose accumulation is also compatible with a stress-protective response because this disaccharide can contribute to the stabilization of proteins and membranes under adverse conditions [44]. In the present system, however, the persistence of severe membrane damage and water loss indicates that these compensatory responses were insufficient to restore homeostasis.

The simultaneous depletion of sucrose, D-glucose, fructose, galactinol, and ascorbic acid at 24 h provides a direct metabolic link between photosynthetic inhibition, carbon limitation, and redox imbalance. Lower soluble-sugar pools are consistent with restricted carbon assimilation, increased respiratory demand, and possible metabolite loss from permeabilised cells. Essential-oil injury is therefore unlikely to be limited to a nonspecific external ‘burning’ effect. For example, *Origanum vulgare* essential oil profoundly altered glutamate, aspartate, and photorespiratory metabolism in Arabidopsis seedlings, demonstrating that volatile mixtures can propagate their effects through central carbon and nitrogen pathways [11]. In the present study, the reduction in ascorbate-related metabolites, together with the strong accumulation of MDA, further suggests consumption or failure of antioxidant buffering as oxidative damage progressed [45]. The late metabolic phenotype is therefore best interpreted as the combined outcome of carbon starvation, osmotic imbalance, oxidative stress, proteolysis, and structural injury to the membrane.

The DSPC analysis provides an additional systems-level view of this progression. The highly interconnected network at 6 h is consistent with coordinated metabolic adjustment during the initial stress response, in which amino acids, organic acids, sugars, and antioxidant-related metabolites remain strongly coupled. Metabolite correlation networks can complement changes in individual metabolite abundance by revealing condition-dependent associations and potentially coordinated metabolic modules [46,47]. Conversely, the markedly reduced 24 h network is consistent with a loss of coordinated covariance among central carbon, nitrogen, and redox-related metabolites as injury became irreversible.

Nevertheless, network density, hub identity, and apparent fragmentation are sensitive to sample size, network-selection thresholds, and data-preprocessing choices [48,49,50]. Given the limited number of biological observations used to construct each time-specific network, the 24 h topology should therefore be described as exploratory evidence consistent with metabolic fragmentation rather than as definitive proof of complete pathway deregulation. Targeted quantification in a larger independent experiment, ideally combined with stable-isotope tracing or metabolic flux analysis, would strengthen this interpretation by distinguishing changes in metabolite pools from changes in pathway activity [51].

Overall, the integration of phenotypic, hydration, membrane, fluorescence, pigment, and metabolomic data supports a coherent mode-of-action sequence in which early membrane destabilisation and oxidative injury are followed by tissue dehydration, photosynthetic dysfunction, and extensive metabolic perturbation. The late accumulation of amino acids, GABA, proline, and trehalose appears to reflect an attempted but ultimately insufficient stress response to progressive structural damage. Importantly, the present study was designed to investigate the phytotoxic activity and physiological mode of action of *S. chamaecyparissus* EO under controlled conditions rather than to benchmark its agronomic efficacy against currently available herbicides. The absence of a positive herbicidal control therefore precludes direct conclusions regarding its competitiveness with synthetic contact herbicides or commercial bioherbicides. Future comparative experiments should include reference herbicides and address formulation, dose optimisation, crop selectivity, environmental safety, and performance under greenhouse and field conditions.

## 5. Conclusions

The present study provides an integrated physiological, biochemical, biophysical, and metabolomic characterisation of the post-emergence phytotoxic activity of *Santolina chamaecyparissus* essential oil against *Bidens pilosa*. Under the controlled experimental conditions used here, the EO induced rapid and concentration-dependent injury, with an ED_50_ of 0.82% (*v*/*v*), followed by progressive tissue dehydration and extensive foliar damage.

The temporal integration of the measured responses supports a mode of action in which early membrane destabilisation and oxidative injury represent central events. Increased electrolyte leakage and lipid peroxidation were followed by impairment of PSII functionality, reduced electron transport, pigment degradation, and extensive reorganisation of primary metabolism. The depletion of soluble sugars, organic acids, and antioxidant-related metabolites, together with the accumulation of proline, GABA, trehalose, and branched-chain amino acids, indicates that the initial contact injury ultimately develops into widespread metabolic dysfunction.

These findings extend the existing evidence on the phytotoxic potential of *S. chamaecyparissus* EO and provide a mechanistic basis for further investigation of its potential as a post-emergence bioherbicidal agent. However, the results refer to a single chemically characterized EO batch and to experiments conducted under controlled conditions. Its practical agronomic potential therefore remains to be established. Future studies should assess batch-to-batch variability, optimise formulation and application strategies, compare its efficacy with reference herbicides, evaluate crop selectivity and environmental safety, and validate its performance under greenhouse and field conditions.

## Figures and Tables

**Table 1:** Chemical composition and metabolite characterization of Santolina essential oil. Compounds are listed in order of their elution on the capillary column. Retention times (RT) are expressed in minutes, and experimental Retention Indices (RI) were determined relative to a series of n-alkanes. Relative chromatographic abundance is reported as the mean uncorrected percentage of the total integrated peak area ± standard deviation (SD) from replicate injections (*n* = 4). These values were not corrected using compound-specific relative response factors and therefore represent relative peak-area abundances rather than absolute concentrations or mass percentages. Structural classification distinguishes between aliphatic chains, monoterpenes, sesquiterpenes, diterpenes, and heterocyclic nitrogenous compounds (pyridines and pyrazines).

RT	RI	Metabolite	Uncorrected Relative Peak Area (%) ± SD	Chemical Class
2.855	706	2-Methyl-1-butanol	0.37% (±0.05)	Aliphatic alcohol
3.638	738	β-Methylpropyl ethanoate	0.01% (±0.00)	Aliphatic ester
5.002	821	1,3-Octadiene	0.05% (±0.01)	Aliphatic alkene
8.729	911	Santolinatriene	0.03% (±0.01)	Monoterpene hydrocarbon
9.443	937	α-Pinene	0.07% (±0.01)	Monoterpene hydrocarbon
10.457	969	3-Alkylpyridine	2.10% (±0.23)	Pyridine derivative (Alkaloid)
11.444	1003	α-Phellandrene	6.15% (±0.40)	Monoterpene hydrocarbon
11.718	1021	4-Carene	0.09% (±0.01)	Monoterpene hydrocarbon
11.945	1031	Limonene	5.87% (±0.14)	Monoterpene hydrocarbon
12.187	1049	Hexyl 2-methylbutanoate	1.14% (±0.08)	Aliphatic ester
12.385	1060	*p*-Menth-1,4-dien-7-ol	1.43% (±0.14)	Oxygenated monoterpene
12.428	1063	Tetramethylpyrazine	5.23% (±0.30)	Pyrazine derivative (Alkaloid)
12.483	1065	Artemisia alcohol	1.13% (±0.07)	Oxygenated monoterpene
12.801	1092	Isopinocamphone	4.32% (±0.19)	Oxygenated monoterpene
12.876	1098	Artemisia ketone	1.04% (±0.20)	Oxygenated monoterpene
13.153	1121	Limonene epoxide	1.09% (±0.09)	Oxygenated monoterpene
13.430	1143	*cis*-Pinocarveol	5.86% (±0.15)	Oxygenated monoterpene
13.761	1173	Dihydrocarveol	1.74% (±0.12)	Oxygenated monoterpene
13.884	1182	*trans*-β-Ocimene	1.40% (±0.05)	Monoterpene hydrocarbon
14.231	1228	*cis*-Carveol	6.10% (±0.09)	Oxygenated monoterpene
14.453	1257	Isobornyl acetate	4.03% (±0.73)	Oxygenated monoterpene (Ester)
14.893	1273	Cuminic alcohol	7.09% (±0.74)	Oxygenated monoterpene (Aromatic)
15.041	1277	Piperitone oxide	1.23% (±0.13)	Oxygenated monoterpene
15.652	1393	β-Bourbonene	0.02% (±0.02)	Sesquiterpene hydrocarbon
15.721	1403	Caryophyllene	0.09% (±0.00)	Sesquiterpene hydrocarbon
15.969	1463	γ-Curjunene	1.03% (±1.10)	Sesquiterpene hydrocarbon
16.102	1491	α-Amorphene	4.18% (±0.14)	Sesquiterpene hydrocarbon
16.197	1492	*cis*-β-Elemenone	4.03% (±0.25)	Oxygenated sesquiterpene
16.287	1504	epi-Bicyclosesquiphellandrene	1.47% (±0.10)	Sesquiterpene hydrocarbon
16.754	1578	α-Cedrene epoxide	2.20% (±0.18)	Oxygenated sesquiterpene
17.351	1663	2-Z-Tridecenol	3.00% (±0.53)	Long-chain aliphatic alcohol
17.589	1704	Cladiell-11-en-7-ol	2.23% (±0.23)	Diterpene alcohol

## Data Availability

The original contributions presented in this study are included in the article/Supplementary Material. Further inquiries can be directed to the corresponding author.

## Supplementary Materials

The following supporting information can be downloaded at: https://www.mdpi.com/article/10.3390/biology15181606/s1, Table S1: Untargeted GC–MS metabolomics dataset and statistical analyses.

## Abbreviations

The following abbreviations are used in this manuscript:

ANOVA	Analysis of Variance
AU/a.u.	Arbitrary Units
Car	Total Carotenoids
Chl a	Chlorophyll a
Chl b	Chlorophyll b
Ctrl	Control
DSPC	Debiased Sparse Partial Correlation
DW	Dry Weight
ED_50_	Effective Dose causing 50% visual phytotoxic injury
EI	Electron Ionization
EL	Relative Electrolyte Leakage
EO	Essential Oil
EOT	Essential Oil Treatment
ETR	Electron Transport Rate
F_0_	Minimum fluorescence in dark-adapted leaves
F_m_	Maximum fluorescence in dark-adapted leaves
F_v_	Variable fluorescence
F_v_/F_m_	Maximum quantum yield of Photosystem II
$F_{m_{Lss}}$	Maximum fluorescence measured under light at steady state
F_Lss_	Steady-state fluorescence under actinic light
F_v_′/F_m_′	Maximum quantum efficiency of Photosystem II in the light-adapted state
FW	Fresh Weight
GABA	γ-Aminobutyric Acid
GC–MS	Gas Chromatography–Mass Spectrometry
HSD	Honestly Significant Difference
LED	Light-Emitting Diode
LHCII	Light-Harvesting Complex II
MDA	Malondialdehyde
MSTFA	N-Methyl-N-(trimethylsilyl)trifluoroacetamide
NIST	National Institute of Standards and Technology
NPQ	Non-Photochemical Quenching
PAR	Photosynthetically Active Radiation
PC1	First Principal Component
PC2	Second Principal Component
PCA	Principal Component Analysis
PLS–DA	Partial Least Squares–Discriminant Analysis
PSI	Photosystem I
PSII	Photosystem II
QC	Quality Control
qP	Photochemical Quenching Coefficient
QY	Effective quantum yield of Photosystem II under light conditions
QY_max_	Maximum quantum yield of Photosystem II
Q^2^	Cross-validated predictability index
R^2^	Coefficient of determination
Rfd	Fluorescence Decline Ratio
RI	Retention Index
RT	Retention Time
RWC	Relative Water Content
SD	Standard Deviation
TBA	Thiobarbituric Acid
TCA	Trichloroacetic Acid
TW	Turgid Weight
UV–Vis	Ultraviolet–Visible
VIP	Variable Importance in Projection
ΦPSII	Effective quantum yield of Photosystem II
